# Medical cannabis authorization and the risk of cardiovascular events: a longitudinal cohort study

**DOI:** 10.1186/s12872-021-02229-6

**Published:** 2021-09-10

**Authors:** Arsene Zongo, Cerina Lee, Jason R. B. Dyck, Jihane El-Mourad, Elaine Hyshka, John G. Hanlon, Dean T. Eurich

**Affiliations:** 1 Faculty of Pharmacy, Université Laval, Quebec City, QC Canada; 2 Population Health and Optimal Health Practices Research Unit, CHU de Québec - Université Laval Research Centre, 1050 Chemin Ste-Foy (office J0-08), Quebec City, QC G1S 4L8 Canada; 3 School of Public Health, University of Alberta, Edmonton, AB Canada; 4 Cardiovascular Research Centre, Department of Pediatrics, Faculty of Medicine and Dentistry, University of Alberta, Edmonton, AB Canada; 5 Department of Anesthesia, St. Michael’s Hospital, University of Toronto, Toronto, ON Canada; 6 Department of Anaesthesiology and Pain Medicine, University of Toronto, Toronto, ON Canada

**Keywords:** Longitudinal cohort study, Medical cannabis, Cardiovascular events, Acute coronary syndrome, Stroke, Hospitalization, Emergency department visit

## Abstract

**Background:**

Cannabis is increasingly used for therapeutic purpose. However, its safety profile is not well known. This study assessed the risk of cardiovascular-related emergency department (ED) visit and hospitalization in adult patients authorized to use medical cannabis in Ontario, Canada from 2014 to 2017.

**Methods:**

This is a longitudinal cohort study of patients who received medical cannabis authorization and followed-up in cannabis clinics, matched to population-based controls. The primary outcome was an ED visit or hospitalization for acute coronary syndrome (ACS) or stroke; and secondary outcome was for any cardiovascular event. Conditional Cox proportional hazards regression was used to assess the association between cannabis authorization and risk.

**Results:**

18,653 cannabis patients were matched to 51,243 controls. During a median follow-up of 242 days, the incidence rates for ACS or stroke were 7.19/1000 person-years and 5.67/1000 person-years in the cannabis and controls group, respectively- adjusted hazard ratio (aHR) of 1.44 (95% CI 1.08–1.93). When stratified by sex, the association was only statistically significant among males: aHR 1.77 (1.23–2.56). For the secondary outcome (any CV events), the aHR was 1.47 (1.26–1.72). The aHR among males and females were 1.52 (1.24–1.86) and 1.41 (1.11–1.79), respectively. Tested interaction between cannabis authorization and sex was not significant (p > 0.05).

**Conclusions:**

Medical cannabis authorization was associated with an increased risk of ED visits or hospitalization for CV events including stroke and ACS.

**Supplementary Information:**

The online version contains supplementary material available at 10.1186/s12872-021-02229-6.

## Highlights

- Among the safety concerns of medical cannabis use, there is limited data on the possible increased risk of cardiovascular events associated with the use of cannabis.
- This study is one of the few large epidemiological cohort studies that assesses the risk of cardiovascular CV events associated with the use of medical cannabis among patients in Ontario, Canada - 2014–2017.
- Overall, our results suggest that medical cannabis authorization was associated with a short-term increased risk of emergency department visit and hospitalization for cardiovascular events.

## Background

The number of individuals using cannabis to manage a health condition is increasing despite the lack of conclusive evidence on the efficacy and safety of cannabis for many of the indications for which it is used [1, 2]. In the first half of 2019, approximately 2.7 million Canadians were using cannabis for medical purposes [3]. Cannabis is also the most commonly consumed licit/illicit substance in the world (recreational use) [4, 5]. Because the safety profile of cannabis remains unclear [6], the increasing use of cannabis could have unintended negative consequences for the users, the healthcare systems and public health in general.

Among the safety concerns, the possible increased risk of cardiovascular (CV) events associated with the use of cannabis is of concern [7]. Different mechanisms have been suggested as possible causes of cannabis-related CV risk including a reversible cerebral vasoconstriction triggered by cannabis use (a possible mechanism of stroke) [8], increase in procoagulant proteins [7–9], ischemia by modulating cannabinoid receptors on vascular smooth muscles and human cardiomyocytes [10, 11] arrhythmia, and others [12]. In a systematic review of 116 case reports, 29 observational studies, the authors concluded that while the data are limited (20 of the 29 studies were cross-sectional or case series), there is some suggestion that cannabis use may have negative CV consequences [7]. Of note, the 116 individuals cases were young (mean age was 31 years), and mainly males (81.9%) and they mainly suffered from ischemic strokes or myocardial infarctions [7]. Moreover, most of the studies included non-medical cannabis users. In other studies, however, an association between cannabis use and the risk of CV event was not found [13, 14].

Overall, the current state of evidence is limited to conclude on the CV safety of cannabis. Therefore, this study aimed to assess the risk of CV events associated with the use of cannabis among patients who received medical cannabis authorization in Ontario, Canada. We hypothesized that the medical use of cannabis will be associated with an increased risk of CV events compared to non-use.

## Methods

### Study design

This is a retrospective longitudinal cohort study of adult patients who have been authorized to use cannabis to manage a health condition (the exposure) matched to patients selected from the general population of Ontario who did not receive cannabis authorization. Each patient authorized to use cannabis was matched to up to three controls.

To proceed to control matching, first, an index date is assigned to each patient who is eligible to be selected as control (from the general population) so that the distribution of the eligible controls’ index dates is similar to that of the cannabis patients. Next, baseline characteristics were assessed before or at the index date. Finally, each patient authorized to use cannabis was matched to up to three controls based on age (± 1 years), sex, Local Health Integration Network location, income quartile, and history of health conditions including diabetes, heart disease, chronic obstructive pulmonary disease, asthma, cancer, musculoskeletal issues, neurological issues, pain, behavioral issues, fatigue, malnutrition, and other metabolic diseases. Matching was completed with replacement and thus an unauthorized patient could have been utilized for one or more authorized patients.

Patients authorized to use cannabis and their matched controls were followed from the index date (first date of cannabis authorization for the cannabis cohort and pseudo index date for the controls) until the occurrence of the event of interest, censoring (death or moved out of province), or the end of the study (March 31st, 2017) which ever occurred first. Each patient’s follow-up was estimated from the index date and up to March 31st, 2017 within the administrative data.

### Study population

The study population was Ontario adult patients who received an authorization to access cannabis for medical purposes in a chain of cannabis clinics between April 2014 and March 2017. These clinics offer consultation for cannabis use and follow-up to all patients based on self-referral or physician referral [15]. To be included in the initial matched cohort, patients had to be aged 18 years or over and have been registered as eligible for the Ontario Health Insurance Plan (i.e., residents of Ontario). Patients were excluded if they had invalid or duplicate identifiers. Controls who had any diagnostic codes related to cannabis use during the study period (ICD-10 codes T407 and F12) were excluded.

### Data sources

This study mainly used Ontario administrative health data that served to select the controls and assess the study outcomes and co-variates. The cannabis cohort was selected using data collected in a group of Ontario cannabis clinics. These data were described in a previous paper [15]. Briefly, in the study period (2014–2017), cannabis access for medical use in Canada was conditional on obtaining a medical prescription and administrative authorization (from Health Canada). Thus, all patients in our cannabis cohort were formally authorized to use cannabis. Patients could be referred in the cannabis clinics by other physicians or self-referred. A comprehensive assessment was made during the initial visit and follow-up visits and data were captured electronically with patients’ consent. As these rich clinical data are only available for the cannabis cohort, both the controls and cannabis cohort administrative health data were used to assess the study variables. The Ontario Institute for Clinical Evaluative Sciences (ICES) provided the administrative data. These data include individual data files for each beneficiary, inpatient files, physician billings (inpatient and outpatient physician services) and prescription drug claims [16]. The Ontario Health Insurance Plan (OHIP) contains information on physician services, including diagnostic codes. The Discharge Abstract Database (DAD) and the National Ambulatory Care Reporting System (NACRS) contain all data on hospitalizations and emergency department visits, respectively. For each emergency visit or hospitalization, up to 25 possible diagnoses were registered according to the International Classification of Diseases system—*tenth Revision* (*ICD‐10*). Of these entries, only one indicates the most responsible diagnosis for the visit. The administrative databases were linked using the unique and encrypted patient health insurance number and covered the period from April 24, 2012 to March 31, 2017. We have previously assessed the healthcare utilizations of the cannabis cohort compared to controls using these data [17].

### Outcomes

For primary CV endpoint, we considered emergency department (ED) visits or hospitalizations with a main (primary) diagnostic code for acute coronary syndrome (ACS) or stroke. The following ICD-10 codes were used to assess this outcome in the databases: I20, I21, I24, I60-I64 (see Additional file 1: Appendix 1 for more details).

A secondary outcome was defined as ED visit or hospitalization with a main (primary) diagnostic code for any CV event. The ICD-10 codes I00 to I99 excluding codes I05 to I09, (i.e., chronic rheumatic heart disease) were used to assess this secondary outcome (see Additional file 1: Appendix 1).

### Other variables

Demographic variables included age, sex, nearest census-based neighbourhood income quintile and area of residence (rural versus urban). We also assessed the following existing morbidities in the period going from 2012 to the index date: asthma, diabetes, metabolic disease, CHF, COPD, cancer, musculoskeletal issues, fatigue, pain, behavioural issues and neurological disorders (see Additional file 1: Appendix 2 for ICD-9 and ICD-10 codes used to assess these variables). Finally, as only congestive heart failure was considered in the initial matching, we also assessed the presence of any cardiovascular event as well as the presence of ACS or stroke in the period before the index date (see Additional file 1: Appendix 2 for details on the definitions and ICD-9 and ICD-10 codes used to define these variables) to characterize CV event history.

### Statistical analysis

Descriptive statistics were used to assess the characteristics of the study sample (mean and standard deviation or median for continuous variables; numbers and proportions for categorical variables). Incidence rates of CV events per 1000 person-years and 95% confidence intervals were calculated for each group. For both the primary and secondary outcomes, conditional Cox proportional hazards regressions, that account for the matching, were used to assess the association between cannabis use and the study outcomes. The models were further sequentially adjusted for history of ACS/stroke and for history of any CV event, respectively. Schoenfeld residuals were used to assess the proportional hazards assumption. Hazard ratios (HR) and 95% confidence intervals (95%CI) were derived from each model.

In sensitivity analyses, we stratified each outcome-specific analysis by sex to assess possible sex-differences. We also stratified the analysis according to age (i.e., ≤ 40 years versus > 40 years). We finally tested for interaction between sex and cannabis authorization, and between age and cannabis authorization. For all analyses, a two‐side *P* < 0.05 was considered as statistically significant. The analyses were performed using SAS version 9.4 (SAS Institute, Cary, NC, USA).

## Results

**Fig. 1:**
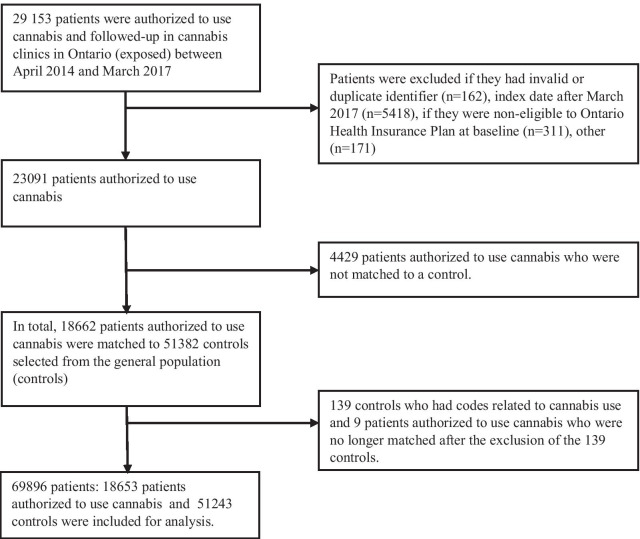
Selection of study population

From 29,153 adult patients who received medical cannabis authorization and were followed-up in the cannabis clinics between 2014 and 2017, 18,653 matched to 51,243 controls were included for analysis (Fig. 1). The majority of patients authorized to use cannabis and the controls were aged 31–60 years and 54% were male (Table 1).

**Table 1:** Characteristics of the study sample

Characteristics	Controls N = 51,243 (%)		Patients authorized to use cannabis N = 18,653 (%)	
*Age, years*				
< 21	331	(0.65)	119	(0.64)
21–30	5578	(10.89)	1972	(10.57)
31–40	10,088	(19.69)	3822	(19.32)
41–50	10,545	(20.58)	4842	(20.49)
51–60	13,227	(25.81)	2858	(25.96)
61–70	7771	(15.16)	1050	(15.32)
71–80	2745	(5.36)	1050	(5.63)
> 81	958	(1.87)	386	(2.07)
*Sex*				
Female	23,206	(45.29)	8528	(45.72)
Male	28,037	(54.71)	10,125	(54.28)
*Nearest census based neighbourhood income quintile*				
1	10,943	(21.36)	4053	(21.73)
2	10,524	(20.54)	3859	(20.69)
3	9943	(19.40)	3595	(19.27)
4	10,327	(20.15)	3726	(19.98)
5	9506	(18.55)	3420	(18.33)
Rural	6046	(11.80)	1798	(9.64)
*Comorbidities considered in the initial matching*				
Asthma	9478	(18.50)	3690	(19.78)
Behavioural disorders	8800	(17.17)	3573	(19.16)
Cancer	4472	(8.73)	1828	(9.80)
Congestive heart failure	295	(0.58)	166	(0.89)
Chronic obstructive pulmonary disease	5722	(11.17)	2351	(12.60)
Diabetes	5390	(10.52)	2214	(11.87)
Fatigue	460	(0.90)	277	(1.49)
Metabolic disease	5945	(11.60)	2605	(13.97)
Musculoskeletal disorders	21,716	(42.38)	8250	(44.23)
Neurological disorders	6812	(13.29)	2886	(15.47)
ED or hospitalization with a main diagnosis code for ACS or stroke before the index date	483	(0.94)	210	(1.13)
History of ACS or stroke before*	3097	6.04	1429	7.66
History of any cardiovascular event**	14,902	(29.08)	6302	(33.79)

CV, cardiovascular; ED, emergency department; OHIP, Ontario Health Insurance Plan; ACS, acute coronary syndrome

^*^includes any ED visit or hospitalization or outpatient visit to physician with a diagnostic code (either primary or secondary) for ACS or stroke

^**^ includes any ED visit or hospitalization or outpatient visit to physician with a diagnostic code for a CV event

The most prevalent morbidities were respectively musculoskeletal disorders (42.87%), asthma (18.83%), behavioral disorders (17.70%), neurological disorders (13.87%) and metabolic diseases (12.23%) (Table 1). Overall, 7.7% of the cannabis users and 6.0% of controls had a history of ACS or stroke (i.e., outpatient, inpatient or ED visit between 2012 and index date with a CV related code, either primary or secondary).

During a median follow-up of 242 days (Q1:113–Q3:401), the incidence rate for the primary outcome (i.e., ED visits or hospitalization with a main diagnosis code for ACS or stroke) was 5.67 (95% CI 4.97–6.46) per 1000 person-years in the control group and 7.19 (95% CI 5.92–8.72) per 1000 person-years in the cannabis group (Q1:113–Q3:401) (Table 2).

**Table 2:** Incidence rates of emergency department (ED) visits or hospitalization for acute coronary syndrome (ACS) or stroke (primary outcome), and for any cardiovascular (CV) event (secondary outcome) among patients authorized to use cannabis and controls

Outcome	Exposition	Number of events	Total person-years	Incidence rates per 1000 person-years (95% CI)
Primary outcome (ACS or stroke)	Patients authorized to use cannabis	102	14,186.68	7.19 (5.92–8.72)
	Controls	223	39,342.55	5.668 (4.97–6.46)
Secondary outcome (any CV event)	Patients authorized to use cannabis	398	14,039.99	28.34 (25.73–31.23)
	Controls	742	39,044.22	19.00 (17.69–20.41)

Patients authorized to use cannabis had an increased risk of ED visit or hospitalization for ACS or stroke compared to controls (adjusted hazard ratio (aHR) 1.44 (95% CI 1.08–1.93) (Table 3).

**Table 3:** Association between medical cannabis authorization and the risk of hospitalization or emergency department (ED) visits for acute coronary syndrome (ACS) or stroke (primary outcome) and for any cardiovascular (CV) event (secondary outcome)

Outcome	Statistical model	Hazard ratio (95% confidence interval)
Primary outcome (ACS or stroke)	Conditional Cox model*	1.48 (1.11–1.97)
	Conditional model further adjusted for prior ACS or stroke and area of living	1.41 (1.05–1.90)
	Conditional model adjusted for history of any CV event and area of living	1.44 (1.08–1.93)
Secondary outcome (any CV event)	Conditional Cox model*	1.52 (1.31–1.77)
	Conditional model further adjusted for history of any CV event and for area of living (rural versus urban)	1.47 (1.26–1.72)

^*^Accounts for the matching that was based on age, sex, income quartile and previous diagnosis of: diabetes, congestive heart failure (CHF), chronic obstructive pulmonary disease (COPD), asthma, cancer, musculoskeletal disorders, neurological disorders, pain, fatigue, behavioural disorders, malnutrition, and metabolic disease

The incidence rates and the hazard ratios for ACS/stroke, stratified by sex and age, are presented in Tables 4 and 5. For these analyses, the aHR was only statistically significant among males (HR: 1.77 (95% CI 1.23–2.56)) and among patients older than 40 years (aHR 1.42 (1.05–1.92)) (Tables 4 and 5). However, the interactions between cannabis authorization and sex, and between cannabis authorization and age were not statistically significant, suggesting that the risks were similar between groups (p-value of interaction was 0.0703 for sex, and 0.6015 for age).

**Table 4:** Sex stratified incidence rates and hazard ratios for emergency department visit or hospitalization for acute coronary syndrome or stroke and for any cardiovascular event among patients with medical cannabis authorisation and non-authorized controls

		Primary outcome: acute coronary syndrome or stroke				Secondary outcome: any cardiovascular event			
Sex	Exposure group	Number of events	Total person-years	Incidence rate per 1000 persons-years (95% CI)	Adjusted Hazard ratio (95% CI)*	Number of events	Total person-years	Incidence rate per 1000 persons-years (95% CI)	Adjusted Hazard ratio (95% CI)*
Males	Authorized to use cannabis	69	7896.50	8.73 (6.91–11.05)	1.77 (1.23–2.56)	233	7819.88	29.79 (26.25–33.81)	1.52(1.24–1.86)
	Controls	132	21,496.29	6.14 (5.18–7.28)	1 [Reference]	416	21,341.38	19.49 (17.72–1.44)	1 [Reference]
Females	Authorized to use cannabis	33	6290.18	5.25 (3.73–7.37)	0.98 (0.59–1.62)	165	6220.11	26.53 (22.82–30.84)	1.41(1.11 -1.79)
	Controls	91	17,846.26	5.10 (4.15–6.26)	1 [Reference]	326	17,702.85	18.41 (16.54–20.51)	1 [Reference]

^*^Conditional Cox model further adjusted for history of any CV and area of living

**Table 5:** Age stratified incidence rates and hazard ratios for emergency department visit or hospitalization for acute coronary syndrome or stroke and for any cardiovascular event among patients with medical cannabis authorisation and non-authorized controls

		Acute coronary syndrome or stroke				Any cardiovascular event			
Age strata	Exposure group	Number of events	Total Person-years	Incidence rate per 1000 persons-years (95% CI)	Adjusted Hazard ratio (95% CI)*	Number of events	Total Person-years	Incidence rate per 1000 persons-years (95% CI)	Adjusted Hazard ratio (95% CI)*
*Age dichotomized*									
≤ 40 years	Authorized to use cannabis	6	4607.47	1.30 (0.58 -2.90)	1.48 (0.32–6.96)	47	4583.87	10.25 (7.72–13.63)	1.34 (0.75–2.42)
	Controls	6	12,200.21	0.49 (0.22–1.09)	Ref	66	12,164.32	5.43 (4.27–6.90)	Ref
> 40 years	Authorized to use cannabis	96	9579.22	10.02 (8.21–12.23)	1.42 (1.05–1.92)	351	9456.13	37.12 (33.50–41.13)	1.47 (1.25–1.73)
	Controls	217	27,142.35	7.99 (7.00–9.13)	Ref	676	26,879.91	25.15 (23.35–27.09)	Ref

The p-value for interaction between age (as dichotomous variable) and cannabis authorization is 0.6015 for the primary outcome and 0.9412 for the secondary outcome

^*^Conditional Cox model further adjusted for history of any CV event and area of living

In our secondary analysis, the incidence rates of any CV event was 19.00 (95% CI 17.69–20.40) per 1000 person-years in the control group and 28.34 (95% CI 25.73–31.23) per 1000 person-years for patients authorized to use cannabis (Table 2). In the model adjusted for history of any CV event, medical cannabis authorization was associated with a significant increased risk of ED or hospitalization for any CV event, aHR: 1.47 (95% CI 1.26–1.70) (Table 3). The incidence rates and the hazard ratios for the secondary outcome, stratified by sex and age, are presented in Table 5. Overall, the risk of CV events was not statistically different among males and females, nor between patients under 40 and those older than 40 years as the interaction terms between sex and cannabis authorization, and between age and cannabis authorization were not statistically significant (p-value for interaction was 0.6209 for sex, and 0.9412 for age).

## Discussion

This longitudinal cohort study suggests that patients authorized to use cannabis had a higher short-term increased risk of ED visits or hospitalizations due to ACS or stroke and due to any CV event in general. When considering stratification by sex and age, the risk of ACS or stroke was only statistically significant among males, and among older patients (> 40 years). However, the interactions between cannabis authorization and sex, and between cannabis authorization and age were not statistically significant, suggesting that the risk was similar between groups.

Our findings are consistent with those of some previous studies suggesting that cannabis use may increase CV risk. A 2017 systematic review of case reports and few observational studies (mainly cross-sectional and including recreational cannabis users) found a possible CV risk with the use of cannabis [7]. More recent studies also suggest an increased cannabis-related CV risk [18, 19]. In fact, evidence suggests that the endocannabinoid system has a significant role in the regulation of cardiovascular system [20, 21]. The activation of cannabinoid receptors 1 and 2 (CB1 and CB2) has effect on blood pressure, heart rate and myocardial contractility [21] that could explain the cannabis-related CV risk. However, not all studies show an association between cannabis use and the CV risk. With some limitations including small sample size, inability to adjust for potential confounders, minimal exposure to cannabis, low-risk profile of the population (young and healthy), cannabis use was not associated with an increased risk of CV events in some studies [14, 22].

The observed similarity of CV risk among males and females is to be interpreted with caution as the lack of statistical power could not be excluded. There is evidence of sex difference in the endocannabinoid system that could differentially affect the cannabis effects among males and females [21]. Research on rodent models of cardiomyopathy showed that the activation of the CB1 receptor triggers cardiomyocyte injury, increases collagen deposition and cardiomyocyte overgrowth whereas activation of CB2 receptors leads to cardioprotective, antifibrotic and antihypertrophic action [23, 24]. A study of the sex differences in the distribution of cannabinoid receptors showed that CB1 receptors are significantly more expressed in the heart of males over 50 years than in the heart of females of the same age group [21]. The opposite was observed for the CB2 receptors [21]. Studies that are specifically powered to detect sex differences in the cannabis-related CV risk are needed.

One of the strengths of our study is the use of one of the largest cohorts of patients with medical cannabis authorization (n = 18,653). This study is one of the few studies that assessed the CV risk among medical cannabis users (most of current studies included non-medical users, who are mainly young and healthy). Our ability to match cannabis patients with population-based controls on a number of important variables also represents a strength of the study.

Among the limitations, we were not able to match all the cannabis cohort patients to at least one control (about 19% were not matched and were excluded from the analysis). This issue has probably led to an underestimation of the CV events as the excluded patients were more likely to be older and had higher rates of morbidities. Moreover, we were not able to account for the concomitant use of drugs that could differentially affect the cardiovascular risk in both groups (drug information was only available for a subset of the study sample). Moreover, residual confounding cannot be refuted because information on variables such as lifestyle parameters (e.g., alcohol, physical activity level, tobacco, body mass index, blood pressure, lipid profile, etc.) are not available in the administrative data. Although we excluded controls who had cannabis-related diagnostic codes during the entire follow-up, there is a possibility that some controls may have used recreational cannabis or self-medicated with cannabis. If present, this misclassification bias would have led to an underestimation of the CV effects of cannabis in our analyses. Confounding by indication may also be a potential limitation for individuals who may have had an underlying condition at the index date, which increased their risk of CV event independently of cannabis exposure. Another limitation is related to a possible high self-referral for younger patients than older. However, the stratification of the analysis by age suggests that this potential bias has not affected the results as the risk was similar between younger and older patients (i.e., the interaction between age and cannabis authorization was not significant). Finally, we were not able to fully assess cannabis exposure as we did not account for the chemical components, cannabis dosing, and the route of administration. Future studies should consider these variables to determine whether the CV risk differs accordingly.

## Conclusion

Overall, this study suggests that there may be increased short-term risk for CV-related ED visit or hospitalization including major events such as ACS and stroke – for patients authorized to use cannabis to manage a health condition. We did not observe a difference in the risk among males and females, nor between younger and older patients. The results can contribute to understand the cardiovascular risk associated with the use of cannabis, particularly for medical purpose.

## Supplementary Information

**Additional file 1**. **Supplemental Table 1.** Definitions of the primary and secondary outcomes.

## Data Availability

The data is not publically available as it combines Ontario administrative data from ICES and clinical data from CCC. Thus, access to the data is contingent to request to both ICES (https://www.ices.on.ca/DAS/Public-Sector/Access-to-ICES-Data-Process) and CCC (https://www.cannabisclinics.ca/).

## Abbreviations

CV: Cardiovascular event
ACS: Acute coronary syndrome
ED: Emergency department
