# Supplementary material for: Medical cannabis authorization and the risk of cardiovascular events: a longitudinal cohort study
Source: BMC Cardiovasc Disord. 2021 Sep 10;21:426. doi: 10.1186/s12872-021-02229-6 (PMC8431905; doi:10.1186/s12872-021-02229-6)
Supplement: Supplementary file 1 — Additional file 1. Supplemental Table 1. Definitions of the primary and secondary outcomes. [file 12872_2021_2229_MOESM1_ESM.docx]

**Appendix Table 1: Definitions of the primary and the secondary outcomes**

|  | ICD-10 codes |
| --- | --- |
| Primary outcome: emergency department visit or hospitalization with a main diagnosis (primary diagnosis code) for any Cardiovascular event (excluding chronic rheumatic heart diseases, ICD-10 I05-I09) | I00-I02 Acute rheumatic fever  I10-I15 Hypertensive diseases  I20-I25 Ischaemic heart diseases  I26-I28 Pulmonary heart disease and diseases of pulmonary circulation  I30-I52 Other forms of heart disease  I60-I69 Cerebrovascular diseases  I70-I79 Diseases of arteries, arterioles and capillaries  I80-I89 Diseases of veins, lymphatic vessels and lymph nodes, not elsewhere classified  I95-I99 Other and unspecified disorders of the circulatory system |
| Secondary outcome: emergency department visit or hospitalization with a main diagnosis (primary diagnosis code) for acute coronary syndrome or stroke | I20 Angina pectoris  I21 Acute myocardial infarction  I24 Other acute ischaemic heart diseases  I60 Subarachnoid haemorrhage  I61Intracerebral haemorrhage  I62 Other nontraumatic intracranial haemorrhage  I63 Cerebral infarction  I64 Stroke, not specified as haemorrhage or infarction |

**Appendix Table 2: Matched authorized versus unmatched authorized cannabis patients**

| Characteristics | Authorized cannabis patients matched to a control (18662) | Authorized cannabis patients not matched to a control (n=4429) |
| --- | --- | --- |
| Age |  |  |
| <21 | 120 (0.64) | 23 (0.52) |
| 21-30 | 1974 (8.55) | 240 (5.42) |
| 31-40 | 3606 (19.32) | 547 (12.35) |
| 41-50 | 3822 (20.48) | 843 (19.03) |
| 51-60 | 4846 (25.97) | 1165 (26.30) |
| 61-70 | 2858 (15.31) | 878 (19.82) |
| 71-80 | 1050 (5.63) | 491 (11.09) |
| >80 | 386 (2.07) | 242 (5.46) |
| Sex (males) | 10132 (54.29) | 2124 (47.96) |
| Rural (yes) | 1798 (9.63) | 519 (11.72) |
| Asthma | 3691 (19.78) | 1737 (39.22) |
| Musculoskeletal disorders | 8256 (44.24) | 3032 (68.46) |
| behavioural disorders | 3582 (19.19) | 2152 (48.59) |
| Cancer | 1828 (9.80) | 1387 (31.32) |
| COPD | 2353 (12.61) | 1706 (38.52) |
| Diabetes | 2215 (11.87) | 1518 (34.27) |
| Fatigue | 279 (1.50) | 1003 (22.65) |
| Metabolic disease | 2609 (13.98) | 2361 (53.31) |
| Neurlogic disorders | 2892 (15.50) | 2146 (48.45) |
| Pain | 615 (3.30) | 1397 (31.54) |
